# Cancer Site-Specific Multiple microRNA Quantification by Droplet Digital PCR

**DOI:** 10.3389/fonc.2018.00447

**Published:** 2018-10-15

**Authors:** Noemi Laprovitera, Maria Grzes, Elisa Porcellini, Manuela Ferracin

**Affiliations:** ^1^Department of Experimental, Diagnostic and Specialty Medicine (DIMES), University of Bologna, Bologna, Italy; ^2^Department of Molecular Biology, Institute of Genetics and Animal Breeding of the Polish Academy of Sciences, Lesznowola, Poland

**Keywords:** microRNA, FFPE (formalin-fixed paraffin-embedded), droplet digital PCR (ddPCR), cancer, EvaGreen chemistry

## Abstract

Archival formalin-fixed paraffin-embedded (FFPE) tissues represent an extraordinary source of smallRNAs, including microRNAs (miRNAs). Contrary to other RNA molecules, miRNAs are stable, nuclease-resistant and quantifiable even in low quality samples. The accurate assessment of miRNA levels in archival samples is of great interest for many pathological conditions, including cancer. In human tumors, microRNA expression is type-specific and can be used as diagnostic, prognostic or response-to-treatment biomarker. In this study, we provide a method for multiple miRNA quantification in 96-well plates, using EvaGreen-based droplet digital PCR technology and miRCURY LNA miRNA assays. This approach allows the absolute quantification of a customizable panel of miRNAs at the same time and under identical experimental conditions, to be used for diagnostic or prognostic applications.

## Introduction

MicroRNAs (miRNAs) are important molecules involved in post-transcriptional regulation of gene expression. Their crucial role in human cancer is well documented (1). MicroRNA expression profiles can distinguish different cancer types and contribute to cancer sub-classification (2). The pattern of miRNA expression can be used to infer the origin site of metastatic tumors (3–5). In addition, the value of microRNAs as molecular biomarkers in formalin-fixed paraffin-embedded (FFPE) specimens and body fluids, and the many applications of miRNA quantification for diagnosis and prognosis in several human cancers were also described (6). MicroRNAs are highly stable molecules that resist to RNAase activity in FFPE specimens, which are the most widely available clinical archival samples, biological fluids, and also microvescicles and can be easily isolated from all these tissues (7–9).

Droplet Digital PCR (ddPCR) is a relatively newborn technology that has been used for several applications, such as rare DNA mutation detection, copy number variation analysis and absolute nucleic acid quantification (10–12). This assessment can be performed using both dsDNA-binding dye EvaGreen-based chemistry or TaqMan probe-based assays.

Droplet digital PCR technology (Bio-Rad patent) consists in sample partitioning into nanoliter-sized water-in-oil droplets, which generates thousands of multiple individual reactions measured as endpoint PCRs. Droplets are then individually analyzed by a fluorescence detector and classified as positive if fluorescence is detected (i.e., the target sequence is included) or negative if not. Finally, the number of positive droplets is used to estimate the target concentration with the application of Poisson correction. Specifically, the real concentration could be underestimated because this technology cannot distinguish droplets with multiple copies of the target molecule. Poisson correction solves this problem, by estimating the number of multiple-target droplets on droplet total number (10, 13).

This powerful technology has many applications in research and diagnostics (12, 14). Droplet digital PCR overcomes the limits of quantitative PCR (qPCR) in performance and accuracy, eluding several problems connected to qPCR methodology, such as the need of reference genes for normalization or replicate samples (15). In a previous study, we assessed the overall precision and accuracy, as well as intra- and inter-assay reproducibility, of EvaGreen-based ddPCR with above standard results (16). Using ddPCR it is also possible to evaluate the expression of specific miRNAs that circulate in biological fluids, including blood (17).

## Methods

### Objectives and validation of the method

In this article we describe a tool for miRNA multi-assay quantification using RNA obtained from FFPE tissues and EvaGreen-based droplet digital PCR technology. Multiple miRNA quantification with ddPCR technology in the same plate has never been performed and could be quite challenging because the optimal annealing temperature and primer amount could change between different miRNA primer sets (16, 18). For multiple miRNA quantification, we had to use the same annealing temperature and primer amount. To achieve this goal, we selected the experimental conditions that were most efficient for the majority of the single assays we tested in the past (18, 19), which were 58°C annealing temperature and 1 μL primer amount. Then, we designed pre-spotted custom plates (96-well format) with 92 different miRCURY LNA miRNA primers (Qiagen, former Exiqon). Using this approach, we were able to assess the expression of 92 different miRNAs at the same time, using the same amount of primer and the same PCR conditions (16, 18).

### Detailed protocol

Here we present a detailed protocol of our approach for multiple miRNA quantification with ddPCR, with the sequential steps necessary to use this method.

#### RNA extraction from FFPE samples

We collected 4–5 tissue slices 10–20 μm thick from 14 diagnostic archival formalin-fixed paraffin embedded (FFPE) blocks from 14 different tumor types. The study was approved by the local ethical committee (Comitato Etico Indipendente dell'Azienda Ospedaliero-Universitaria di Bologna, Policlinico S.Orsola Malpighi). All subjects gave written informed consent in accordance with the Declaration of Helsinki. Pathological characteristics of cancer patients are detailed in Supplementary Table 1.

With the assistance of a Haematoxylin and Eosin (H&E) stained section, we dissected the best tumor area and placed in 2 mL Eppendorf tubes (cat. no. H0030120094).

Total RNA can be isolated using any standard method and commercial kit currently available. We describe herein a protocol for the isolation of RNA from FFPE samples using the RecoverAll Total Nucleic Acid Isolation Kit for FFPE (Ambion/ThermoFisher, cat. no. AM1975). In detail, FFPE tumor sections underwent paraffin removal, by adding 1 mL of 100% xylene and incubating at 50°C for 3 min. After centrifugation, xylene was removed without disturbing the pellet; this was then washed twice with 1 mL of 100% ethanol and left to air dry 45 min at room temperature, to remove any residual ethanol. Protease digestion was performed adding 200 μL of Digestion Buffer and 4 μL of Protease enzyme. Samples were left to incubate for 15 min at 50°C and 15 min at 80°C in heat blocks. The Isolation mix was then prepared, mixing 240 μL of Isolation Additive with 550 μL of 100% ethanol, and added to the sample. All this mixture was then passed through a filter cartridge and collected in a collection tube through centrifugation at 10000 g for 30 s. The filter was then washed firstly with 700 μL of Wash 1 solution and then with Wash 2/3 solution and centrifuge to remove any residual fluid. A mixture containing 6 μL of 10X DNase buffer, 4 μL of DNase and 50 μL of Nuclease free water was added to the center of the filter and left to incubate 30 min at room temperature. After repeating the washes with Wash 1 and Wash 2/3 solutions, filter underwent to the elution of RNA with 60 μL of nuclease-free water.

RNA quality and quantity were assessed by Nanodrop spectrophotometer (ThermoFisher) and frozen at −80°C.

#### cDNA synthesis

The conversion of RNA to cDNA was performed using the Universal cDNA synthesis kit II (Exiqon, cat. no. EX203301PR), following the manufacturer's protocol.

RNA was diluted to a concentration of 5 ng/μL using nuclease-free water. The mixture for reverse transcription was prepared for each sample mixing: 2 μL of 5x Reaction Buffer, 4.5 μL of nuclease free water, 1 μL of enzyme mix, 0.5 μL of synthetic RNA (Sp6) spike-in and 2 μL of diluted RNA (10 ng of total RNA). The reaction was performed in a conventional thermal cycler and comprised an incubation step (60 min at 42°C), enzyme heat inactivation (5 min at 95°C) and a holding step (4°C forever). Resulting cDNA was then conserved at −20°C in 1.5 mL LoBind DNA Eppendorf tubes (cat. no. H 0030 108 051).

#### cDNA dilution

Just before the next steps, cDNA was diluted 1:50. A further dilution of 1:10 (final 1:500) was prepared to assess one very abundant microRNA (miR-21-5p) and the internal control assay UniSP6, in order to avoid the saturation of the positive droplets.

#### miRNA plate setup

Pre-spotted custom plates including 89 different cancer-specific miRNA primer sets were designed using miRCURY LNA Custom PCR Panel (Qiagen, former Exiqon) in the 96-well format (cat. no. 339330, cat. no. 339332). Three additional assays for small non-coding RNAs (SNORD44, SNORD48 e snRNAU6) were included as reference. The remaining wells contained two inter-plate calibrator assays (UniSp3), a control plate assay (UniSP6) and a no template control (NTC) well.

Pre-spotted custom plates have both forward and reverse PCR amplification primers in an amount that would be sufficient for one qPCR reaction. We tested these primers as individual assays (16), and verified that this amount is excessive for EvaGreen-based ddPCR, because of the broad fluorescence amplitude of the negative signal. Half the amount was the best solution for ddPCR testing. Thus, we split the plate primers in two plates, as described in the next step.

#### Droplet generation and PCR

Droplet digital PCR workflow was performed using a miRNA quantification protocol that was recently developed (6, 18) using a 20 μL volume of PCR mix containing 10 μL of 2X QX200 ddPCR EvaGreen Supermix (Bio-Rad, cat. no. 1864034), 6 μL of nuclease-free water and 4 μL of diluted cDNA.

To split into two plates the pre-spotted primers, we re-suspended each well in a double amount of EvaGreen and water and transferred half amount in a second plate (Eppendorf, cat. no. H 0030 128 605). At the end of this step, we obtained two 96-well plates with half amount of primers in a volume of 16 μL, both ready for the addition of the diluted cDNA, which was performed with a multichannel pipet.

Each ddPCR assay mixture was loaded into a disposable DG8 Cartridge (Bio-Rad, cat. no. 1864008) located into a cartridge holder (Bio-Rad, cat. no. 1863051). Then, 70 μL of droplet generation oil for EvaGreen (Bio-Rad, cat. no. 1864005) was loaded into each of the eight oil wells. The cartridge was then covered with a DG8 Gasket (Bio-Rad, cat. no. 1863009) and placed inside the QX200 Droplet Generator (Bio-Rad, cat. no. 1864002). Upon completion of droplet generation, the droplets were carefully transferred to a new Eppendorf blue twin.tec 96-well PCR plate (cat. no. H 0030 128 605). The plate was heat-sealed with a pierceable aluminum foil (Bio-Rad, cat. no. 1814040) into the PX1 PCR Plate Sealer (Bio-Rad, cat. no. 1814000) and placed in a thermal cycler. Thermal cycling conditions were: 95°C for 5 min, then 40 cycles of 95°C for 30 s and 58°C for 1 min and three final steps at 4°C for 5 min, 90°C for 5 min and a 4°C infinite hold. It is mandatory to use a ramping rate of 2°C/second in every step.

#### Droplet reading and data analysis

After PCR is completed, the sealed plate was transferred into the plate holder of the QX200 Droplet Reader (Bio-Rad, cat. no. 1864003). Using QuantaSoft software (Bio-Rad), the analysis was set up and started in order to analyze the droplets with an optical detector. At the end of the plate reading, the resulting data were analyzed with QuantaSoft software v1.7. Specifically, from the 2D amplitude plot, the positive droplets in each well were selected using the lasso tool. This function allows to manually select the positive cloud by drawing a circle around it, and finally obtain the miRNA amount (copies/μL). Using the events tab, the number of positive and total generated droplets were evaluated. The general performance of EvaGreen ddPCR consented to obtain a total number of 18,000–21,000 droplets per well.

## Results

### Reproducibility

**Figure 1:**
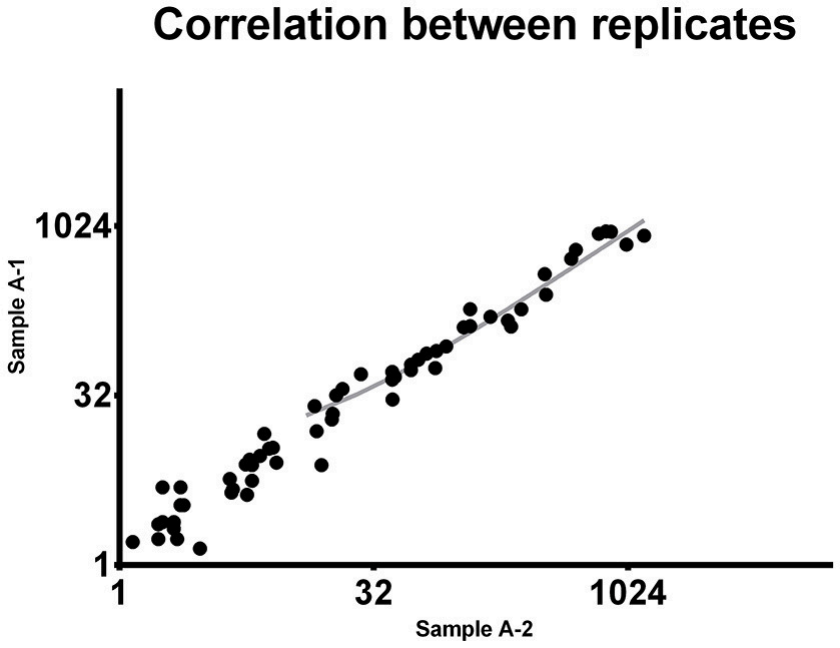
Correlation between replicates. The same 92-miRNA panel was assessed twice in the same sample. Results were highly concordant (Pearson *r* = 0.9537). Each dot represents a microRNA. The miRNA absolute expression is presented as copies per microliter of the amplification reaction mixture. Axis scale is log2 transformed.

To demonstrate the reproducibility of this multiple quantification approach, the same sample was assessed twice using the procedure above described. Pearson correlation analysis was performed to assess the correlation between replicates. Comparing the miRNA expression in the two samples the *p*-value resulted highly significant (*P* < 0.0001). Pearson *r* was found to be 0.9537 (0.92–0.97 95% confidence interval), indicating a strong reproducibility of the methodology (Figure 1).

### Application and effectiveness

Using this multiple miRNA quantification tool, we evaluated the miRNAs expression of 14 FFPE samples from different tumor types (liver, skin, breast, gastric, endometrium, testis, GI-neuroendocrine, prostate, urothelial, kidney, colon, pancreas, lung, ovary).

Our custom plate was designed to contain the most cancer-specific miRNAs: specifically, 89 cancer-specific miRNAs and 3 reference genes.

**Figure 2:**
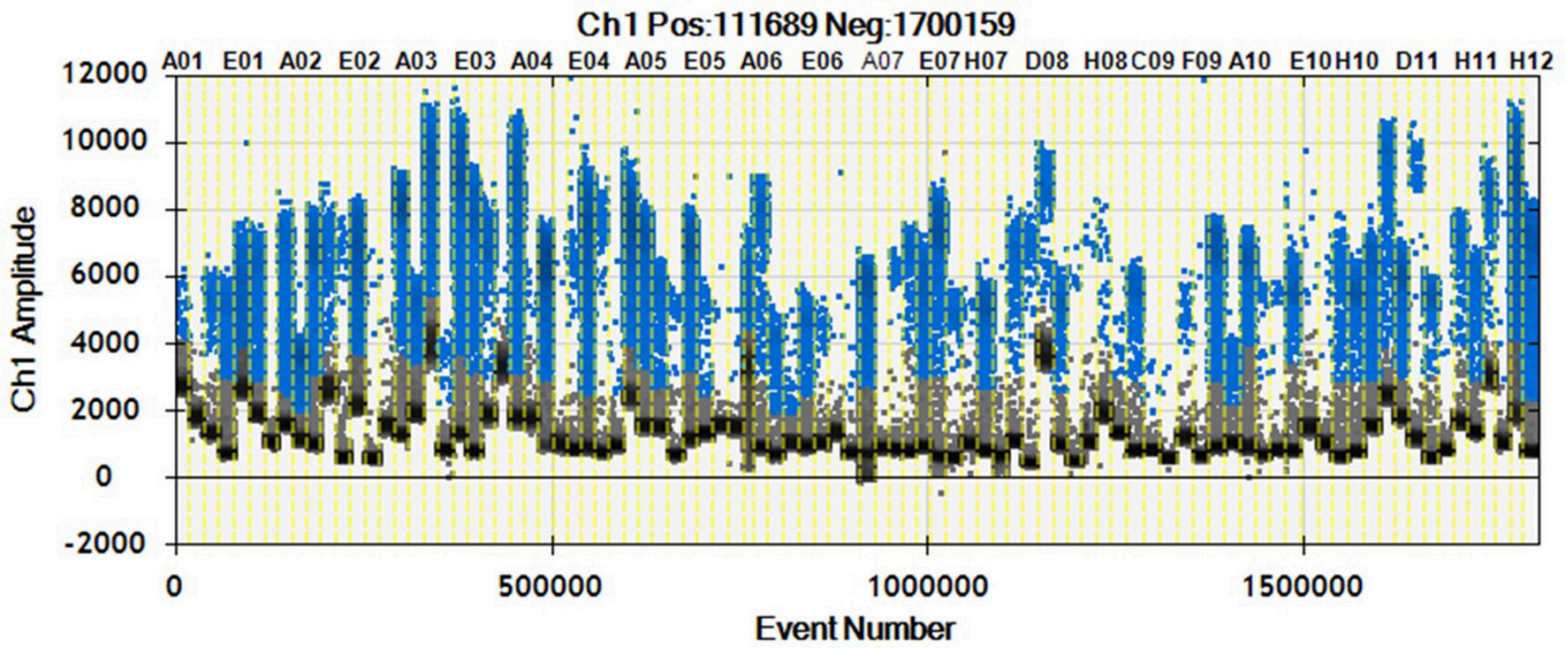
1D Plot representation. Droplet distribution for different miRNA assays evaluated in the same sample. 1D Plot representation shows positive (blue) and negative (black) droplet amplitudes. The fluorescence amplitude can change according to the assay.

**Figure 3:**
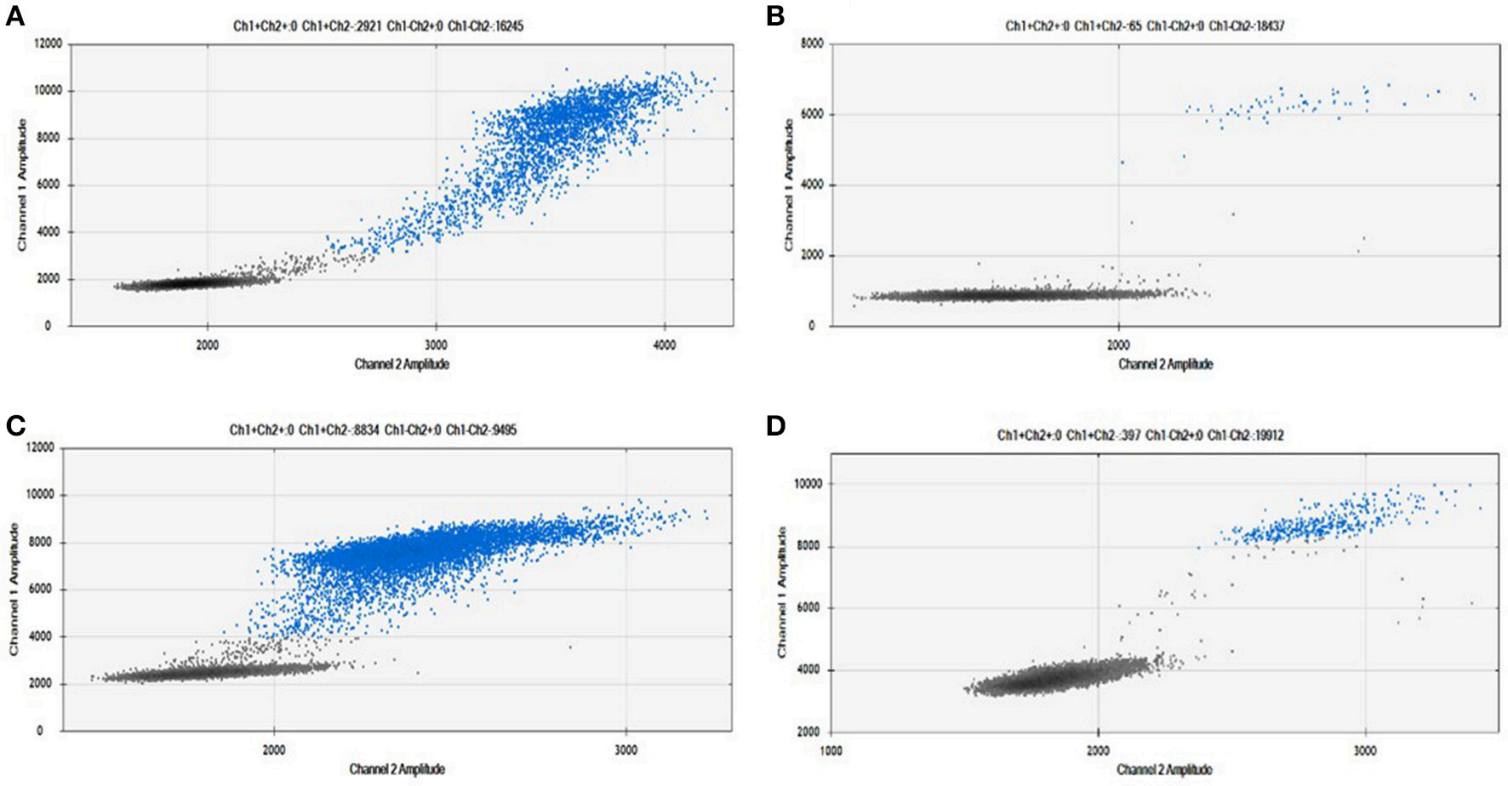
Positive droplet selection in 2D Plot. Bi-dimensional droplet plots of four illustrative miRNA assays: **(A)** RNU6, **(B)** miR-149-5p, **(C)** miR-24-3p, and **(D)** miR-210-3p. Droplet clouds could have different appearance and shapes, but droplet positive selection is always possible.

As shown in Figure 2, we were able to obtain a good separation between positive and negative droplets for all the miRNA assays in our panel. The above detailed experimental conditions allowed a reliable and efficient quantification of all the targets in the same experiment. In Figure 3 we represented some 2D plots of different miRNA assays: the shape of the positive clouds could vary between targets, but it is still easily selectable with the above mentioned QuantaSoft lasso tool.

**Figure 4:**
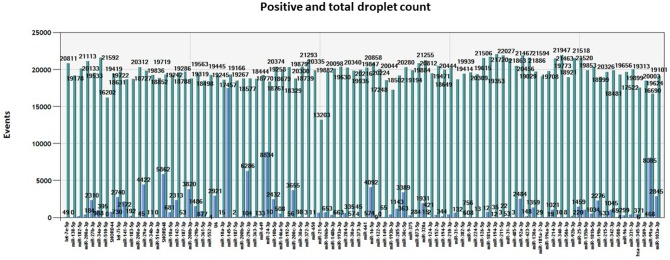
Number of events. Total number of positive (blue) and negative (green) droplets visualized in QuantaSoft software (Bio-Rad) for each miRNA assay assessed in the same tumor sample.

We obtained an average total number of droplets of 20,000 (Figure 4), thus confirming the high sensitivity of the method and accurate detection even of rare targets.

**Figure 5:**
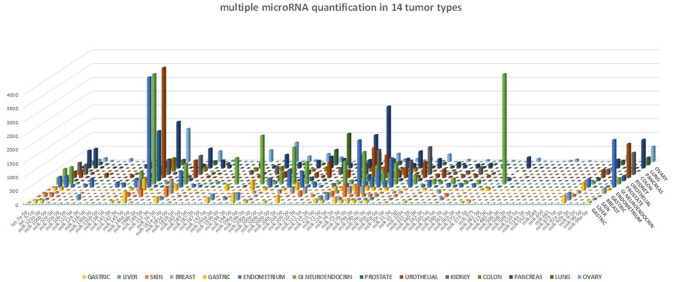
miRNA profile in human cancers. The 89-miRNA expression profile (normalized on reference genes) across 14 different cancer types, as detected by our ddPCR multi-assay method. Each tumor displays a specific miRNA expression profile.

One of the many applications of this tool is represented by the analysis of miRNA expression profile in cancer. By analyzing different cancer types, we were able to validate the expected miRNA signatures, in agreement with our previous data obtained with microarray technology (4). The miRNA profile of 14 different tumor samples is shown in Figure 5. Although we could not directly compare the miRNA quantification provided by a probe hybridization-based technology (Agilent microRNA microarray) and a digital PCR technology, we analyzed the correlation in microRNA quantification between these two approaches, after data normalization, and observed a highly significant correlation of the data (*p* < 0.0001, Spearman *r* > 0.7) for all the tested cancer types.

## Discussion

### Advantages and limitations

Our miRNA multi-assay tool suits the need for a simultaneous absolute quantification of different miRNA targets. The detection of up to 92 different targets all at once and in the same ddPCR experiment, consents to adjust the use of this method for any user need.

We demonstrated that using EvaGreen based-ddPCR and our PCR conditions the quantification of all targets was efficient and reproducible, despite starting from low quality material as FFPE tissue. This novel approach can be particularly appropriate when the miRNAs are expressed at low levels and wouldn't be detected by standard quantitative PCR. Other studies have already proved ddPCR superior accuracy, which overcomes the need of replicates and reduce the experimental costs (15, 16).

Since this tool provides the quantification of up to 92 miRNAs per experiment, it is necessary to identify a focused custom panel of interest. It is important to pay attention to the step of droplet selection during the analysis, and carefully distinguish the positive from the negative droplets. This is a critical step and must be done analyzing each well one-by-one in the 2D amplitude plot and selecting the positive cloud manually with the available software tools.

Even if in some cases the separation could be further optimized (e.g., changing the annealing temperature or the amount of primer), for all the assays that were tested in our experiment a good separation between positive and negative droplets was obtained.

The method described in this paper represents a remarkable innovation. Indeed, the multiple miRNA quantification with ddPCR in the same experiment has never been described. In addition, the possibility to extend this approach to archival samples could support and improve disease biomarker discovery and validation.

Given the sensibility and flexibility of this method, possible applications include: discovery or validation of miRNA biomarkers; miRNA quantification in every type of human tissue, including FFPE, biological fluids and fresh tissue; absolute quantification of multiple miRNAs at the same time in subcellular compartments (e.g., exosomes and microvescicles).

## Author contributions

NL, MG, and EP carried out the experiments and drafted the initial manuscript. MF reviewed the results and wrote the paper. All authors reviewed and approved the final manuscript as submitted.

### Conflict of interest statement

The authors declare that the research was conducted in the absence of any commercial or financial relationships that could be construed as a potential conflict of interest.
